# Mitigation of salinity stress in Sesbania through inter-cropping with halophyte *Hedysarum scoparium* in dry-land conditions

**DOI:** 10.3389/fpls.2026.1756353

**Published:** 2026-03-11

**Authors:** Ahmad Azeem, Mai Wenxuan

**Affiliations:** State Key Laboratory of Desert and Oasis Ecology, Xinjiang Institute of Ecology and Geography, Chinese Academy of Sciences, Urumqi, China

**Keywords:** dry-land, fodder, halophytes, inter-cropping, saline irrigation

## Abstract

Salinity severely limits fodder crop productivity in dry-land regions by inducing ionic imbalance, osmotic stress, oxidative damage, and metabolic dysfunction. This study evaluated the growth, physiological, and biochemical responses of *Sesbania sesban* (L.) Merr. under mono-cropping and inter-cropping systems with halophyte *Hedysarum scoparium* Fisch. et Mey. (HS). The crops were grown under both saline and fresh water irrigation. Inter-cropping with HS significantly alleviated salinity stress in Sesbania by improving ionic balance and reducing stress-related metabolite accumulation. Under saline conditions, inter-cropped Sesbania showed higher chlorophyll content, enhanced photosynthetic pigments (chlorophyll *a*, chlorophyll *b*, carotenoids, and chlorophyll *a*/*b* ratio), and improved growth traits, including plant height, biomass per plant, and total fodder yield, compared with mono-cropped plants. Furthermore, inter-cropping increased stress-responsive proteins and antioxidant enzyme activities, particularly catalase and ascorbate peroxidase, resulting in reduced hydrogen peroxide and malondialdehyde levels. Overall, inter-cropping Sesbania with HS reduces ionic and oxidative stress, leading to improved physiological performance. Consequently, this system offers a sustainable and cost-effective strategy for enhancing fodder production in saline dry-land environments.

## Introduction

Soil salinity and aridity are major constraints to agricultural production worldwide, particularly in dry-land regions. In these areas, low precipitation, high evapotranspiration rates, poor soil structure, and marginal water quality severely limit crop growth and sustainability (Ahmed et al., 2022; Murtaza Arain et al., 2024). Globally, approximately 800 million hectares of agricultural land are affected by salinity, including 20%–25% of irrigated agricultural areas (Singh, 2021). This problem is further intensified in dry-land regions due to climate change and increasing pressure on freshwater resources (Azeem, Mai et al., 2024a, Sofyan et al., 2026). In South Asia, particularly in Pakistan, dry-land regions such as Tharparkar are increasingly affected by soil salinization and water scarcity. These conditions severely limit conventional crop production due to saline soils and limited freshwater availability (Azeem, Mai et al., 2024b, Astiko et al., 2025). Despite these constraints, the region possesses abundant saline water resources and sandy soils with substantial yet underutilized agricultural potential (Hadi et al., 2018; Hassani et al., 2020; Azeem and Mai, 2024). This potential may be harnessed through targeted remediation strategies, including saline water-based precision irrigation. Additional measures include the introduction of halophytic plant species and salt-tolerant crops, as well as the improvement of soil properties using organic and inorganic amendments (Hadi et al., 2018; Hassani et al., 2020; Azeem and Mai, 2024). Among halophytic species, *Hedysarum scoparium* Fisch. et Mey. (HS) has attracted increasing attention due to its strong tolerance to salinity and drought and its ability to improve soil properties in arid environments (Zuo et al., 2020; Al-Tamimi et al., 2023). HS produces high biomass under saline conditions and is widely used in land rehabilitation (Heikal et al., 2025). This is attributed to its capacity to enhance soil physical, chemical, and biological properties, thereby creating a more favorable growth environment for companion crops in inter-cropping systems (Li et al., 2019). In addition, HS can influence soil microbial communities under saline stress by enhancing rhizosphere activity, thereby promoting nutrient cycling and soil health (Chen et al., 2021). The incorporation of HS into dry-land agroecosystems may facilitate salinity stress alleviation and promote improved productivity of inter-cropped species. *Sesbania sesban* (L.) Merr. is a fast-growing annual shrub indigenous to the arid and semi-arid regions of Pakistan and India (Khan et al., 2022, Azeem, Mai et al., 2024c). The widespread cultivation of Sesbania under rain-fed agriculture is attributable to its pronounced inherent tolerance to abiotic stressors, including drought, salinity, and elevated temperature regimes (Khan et al., 2022; Wu et al., 2024). Beyond its resilience, this species functions as a green manure crop internationally, providing substantial benefits for soil fertility through nitrogen fixation and the incorporation of organic matter (Nikam et al., 2024; Yu et al., 2025).

Numerous studies have described that growing halophytic plants in sandy soil under saline irrigation can improve plant performance under dry-land conditions (Cortinhas et al., 2020; Liu et al., 2022). Such approaches have been shown to enhance soil quality and crop productivity even when saline water is used for irrigation (Azeem, Mai et al., 2023a, Ibarra-Villareal et al., 2025). In Mediterranean and temperate regions, halophyte-based inter-cropping systems have been reported to reduce soil sodium and chloride and to lower electrical conductivity (EC), thereby mitigating soil salinity (Almas et al., 2021; Hamed et al., 2021; Castro-Camba et al., 2022). Inter-cropping halophytes with commercial crops has also improved crop yield and quality, such as increased biomass and seed yield in cotton and enhanced vitamin C content in cauliflower under saline conditions (Mai et al., 2023; Sogoni et al., 2025).

However, the mechanisms underlying salinity stress alleviation by halophyte inter-cropping in fodder crops remain poorly understood (Dai et al., 2025). In particular, limited information is available on the role of HS in regulating ionic balance, oxidative stress, and growth responses of companion fodder crops in dry-land agroecosystems, representing a critical knowledge gap (Thompson et al., 2025). To address this gap, the present study evaluated the effects of mono-cropping and inter-cropping systems on the growth performance of Sesbania and halophyte HS under saline irrigation in a dry-land environment. Furthermore, this study compared the growth responses of both species under mono-cropping and inter-cropping conditions. It also assessed the role of halophyte-assisted inter-cropping in sustaining the growth of salt-tolerant fodder crops under saline stress. In addition, the effects of inter-cropping on plant growth, development, and productivity under saline conditions were examined. These findings provide a scientific basis for the optimized use of halophytic plant resources and the development of sustainable land reclamation strategies.

## Materials and methods

These field experiments were conducted at the Sindh Engro Coal Mining Company experimental site near Islamkot, coal block II, in Tharparkar, Sindh, Pakistan. In this study, two plant species were used: *S. sesban* (L.) Merr. and HS. The seeds of Sesbania were purchased from the local market in Pakistan, whereas the seeds of HS were imported from China for this experimental study. A mono-cropping experiment was conducted from 19 June 2023 to 10 October 2023 for Sesbania and from 10 November 2023 to 13 March 2024 for HS. The inter-cropping experiment was conducted from 8 November 2023 to 10 March 2024. The Tharparkar experimental site is characterized by extreme weather conditions, including prolonged hot summers with temperatures reaching up to 50°C and short winters with minimum temperatures of approximately 7°C (Azeem, Mai et al., 2025a). The region receives low annual rainfall of approximately 100 mm, while the annual evaporation rate is high, reaching approximately 2,600 mm. The soil at the experimental site is sandy, characterized by low nutrient content and minimal water holding capacity, which limits crop growth under dry-land conditions. The physicochemical properties of the soil, including EC, texture, pH, organic carbon, nitrate (NO_3_), ammonium (NH_4_), and chloride (Cl) concentrations, are shown in **Table 1**.

**Table 1:** Soil physical and chemical properties.

EC (dS/m)	Texture	Organic carbon (%)	pH	NO_3_ (mg/kg)	NH_4_ (mg/kg)	Cl (mg/kg)
0.167	Loamy sandy	0.30	6.54	260.326	0.012	4.277

EC, electrical conductivity; NO_3_, nitrate; NH_4_, ammonium; Cl, chloride.

### Experimental design

In both the mono-cropping and inter-cropping experiments, each plot had an area of approximately 37.16 m^2^, with a plant-to-plant spacing of 0.3 m and a row-to-row spacing of 0.76 m. There were three replicates of each treatment in each experiment. Healthy seeds of both plant species were selected for the experiment to ensure uniformity among treatments. This study was designed using a completely randomized block design, and seeds were planted in the experimental field according to the assigned treatment. Urea was applied at a rate of 242 kg/ha before planting to minimize nitrogen deficiency under saline conditions, where nutrient uptake is often impaired. The same nitrogen rate was maintained across all treatments to avoid confounding salinity effects with nitrogen availability (Azeem, Mai et al., 2024a). In this study, two irrigation treatments were applied: control (fresh water) and saline water. During the initial 20 days after sowing, all treatments received fresh water to support seedling establishment. After this period, once the seedlings were well established in the field, the saline water treatment was initiated. **Supplementary Figure S1** describes the experimental design (B) and field site location (A). The irrigation water used in the study differed in salinity and chemical composition. Key physical and chemical parameters, including EC, total dissolved solids (TDS), nitrate, nitrite, chloride, fluoride, and manganese concentrations of both control and saline water, are summarized in **Table 2** (Azeem et al., 2024d).

**Table 2:** Water quality parameters.

Water quality	EC (dS/m)	Total dissolved solids (mg/L)	Nitrate (mg/L)	Nitrite (mg/L)	Chloride (mg/L)	Fluoride (mg/L)	Manganese (mg/L)
Saline water	4.368	6,240	0.4	0.002	2,231.03	1.15	0.006
Fresh water	0.357	510	0.4	0.015	196.75	0.35	0.006

EC, electrical conductivity.

These experiments were irrigated using a drip irrigation system. A water meter was installed in the drip irrigation system to measure the amount of water applied. Fertilizer was applied to the plants during the experimental period through a drip irrigation system. Each emitter on the drip line had a discharge rate of 3 L/h. The drip irrigation system was operated 15 minutes per day for both water treatments (control and saline), providing a uniform irrigation rate of approximately 6 mm/day. The watering was performed every day.

### Growth traits

At the end of the experiment, plants of both plant species from each treatment and each replicate were harvested. Plant height and root length were measured using a measuring tape. Fresh shoot and root weights were determined using an analytical balance to calculate total fresh weight per plant for each replicate. Fresh weight per plant was used to calculate yield per acre by multiplying the mean plant weight by plant density (11,040 plants/acre).

### Leaf chlorophyll content and chlorophyll fluorescence

At the end of experiments on 10 October 2023, and 10 and 13 March 2024, the leaf chlorophyll content (SPAD) values of each plant species were measured for each replicate under both planting methods (mono- and inter-cropping). Measurements were taken using a portable chlorophyll meter (SPAD-502 Plus; Oakoch OK-Y104, Shenzhen, China).

Chlorophyll fluorescence was measured using a PAM-2500 fluorometer (Walz, effeltrich, Germany). Fully developed leaves were selected for measurement using a pulse-amplitude modulation (PAM) fluorometer. The leaves were placed in a dark site for 25 minutes before the measurement. Minimal fluorescence (F_o_) was then measured by applying a weak modulated light (<0.1 µmol photon m^−2^ s^−1^). After that, high-intensity light (10,000 µmol photons m^−2^ s^−1^) was applied for 0.6 s to obtain maximal fluorescence (F_M_). The maximal photochemical efficiency of photosystem II (F_V_/F_M_) was measured using F_o_ and F_M_ (F_V_/F_M_ = F_M_ − F_o_/F_M_). According to the Krall and Edwards method, electron transport rate (ETR) was calculated (Krall and Edwards, 1992) using the following equations.


$${\text{Φ}}_{\text{PSII}}=\;\frac{{\text{F}}_{\text{M}}-{\text{F}}_{\text{o}}}{{\text{F}}_{\text{M}}}$$

$$\text{ETR}={\text{ Φ}}_{\text{PSII}}\times \text{PPFD}\times 0.84\times 0.5$$

where F_M_ is the maximal fluorescence, F_o_ is the minimal fluorescence, Φ_PSII_ is the effective photochemical quantum yield of photosystem II, PPFD is the incident photosynthetic photon flux density (µmol photons m^−2^ s^−1^), 0.84 is the assumed leaf absorptance, and 0.5 represents the fraction of absorbed photosynthetically active radiation allocated to photosystem II.

### Photosynthesis pigments

Fresh leaves of both plant species were ground in cold 80% acetone using a pestle and mortar for each replicate and treatment. The homogenate was centrifuged at 3,000 × *g* to separate the liquid. Photosynthetic pigment concentrations were determined using a UV–visible spectrophotometer (DU-730, Beckman Coulter, new jersey, USA). Absorbance of the pigment extracts was measured at 663.2, 646.8, and 470 nm for the estimation of chlorophyll *a* (CHI_a), chlorophyll *b* (CHI_b), and carotenoids (CAR), respectively. Pigment concentrations were calculated using the equations described by Lichtenthaler (1987).

### Hydrogen peroxide and malondialdehyde content

Fresh leaves from both plant species were homogenized for each replicate and treatment under each planting method in 5 mL of 3.1% trichloroacetic acid. The homogenate was centrifuged at 12,000 × *g* for 14 minutes at 4°C to obtain the extract. Hydrogen peroxide (H_2_O_2_) content in leaf tissues was determined using the potassium iodide (KI) reagent method described by Loreto and Velikova (2001). Malondialdehyde (MDA) content was estimated following the method of Heath and Packer using thiobarbituric acid (TBA) and trichloroacetic acid (TCA) (Heath and Packer, 1968).

### Oxidative stress marker and antioxidant

Antioxidant enzymes were extracted from the leaves of both plant species under all treatments and replicates following the method described by Hameed (Azeem, Mai et al., 2025b). Catalase (CAT) activity was determined by monitoring the decrease in absorbance at 240 nm according to the method of Bardsley et al. (1984). Ascorbate peroxidase (APX) activity was measured at 290 nm following the method of Nakano and Asada (1981). Protein content in leaf extracts was quantified using the Bradford method.

### Ions analysis

Chloride (Cl), ammonium (NH_4_), and nitrate (NO_3_) concentrations in the leaves of both plant species were determined following the method described by Shoukat, using leaf extracts obtained by soaking fresh leaf samples. Ion concentrations in the extracts were subsequently analyzed using an ion-selective electrode coupled with a Bante instrument.

### Statistical analysis

Data normality was assessed using the Shapiro–Wilk test. A three-way analysis of variance (ANOVA) was performed to evaluate the effects of species, planting method, and water treatment on growth traits, photosynthetic pigments, oxidative stress markers, antioxidants, chlorophyll fluorescence parameters, and ion concentrations. The interaction effects among these factors were also evaluated for all dependent variables. Furthermore, Tukey’s test (p < 0.05) was used for multiple comparisons among treatment means, including interaction effects. Correlation heat map and principal component analysis (PCA) were generated using python. All statistical analyses were performed in Python using the packages Scipy.Stats, Statsmodels, and Seaborn.

## Results

### Growth traits

**Figure 1:**
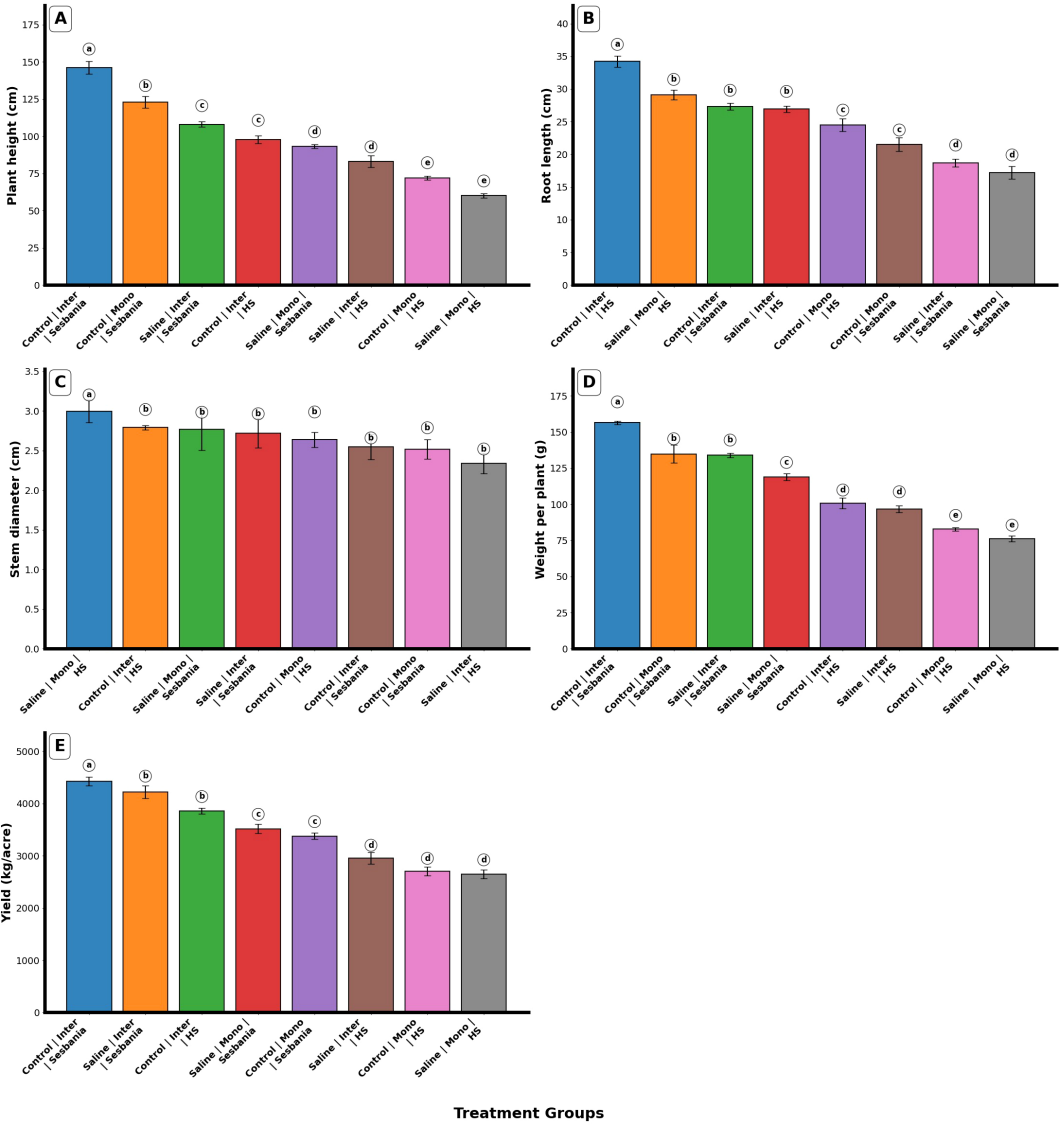
Effects of planting methods and irrigation on growth traits of both plant species: **(A)** plant height, **(B)** root length, **(C)** stem diameter, **(D)** weight per plant, and **(E)** yield. Different letters indicate the significant differences among treatment interactions (p < 0.05). Inter, inter-cropping; Mono, mono-cropping; HS, *Hedysarum scoparium* Fisch. et Mey.

Water treatment and planting method significantly affected the growth traits of both plant species (p < 0.01). Plant height (PH) showed a significant main effect in the single-factor analyses for water treatment, planting method, and species (p < 0.001). Among the interaction terms, only the interaction between water treatment and species was significant (p < 0.05), whereas all other interactions were not significant (p > 0.05). Planting method (mono- and inter-cropping) and saline irrigation had significant effects (p < 0.05) on plant height of both plant species, as shown in **Figure 1**. Sesbania maintained a higher PH than HS under inter-cropping conditions under control and saline irrigation.

Root length (RL) showed a significant main effect in the single-factor analyses for water treatment, planting method, and species (p < 0.001); however, interaction effects showed mixed responses. Among the interactions, water treatment × species (p < 0.01) and water treatment × species × planting method (p < 0.05) were significant. Planting method (mono- and inter-cropping) and saline irrigation had significant effects (p < 0.05) on the RL of both plant species, as shown in **Figure 1**. HS maintained a higher RL than Sesbania under inter-cropping conditions under control and saline irrigation. This may indicate that HS develops a deeper root system under these conditions, which could influence salt distribution in the root zone and indirectly benefit the growth of Sesbania.

According to the three-way ANOVA results, the stem diameter (SD) of both plant species showed no significant main effects (p > 0.05). However, interaction analysis revealed that the SD of HS under mono-cropping with saline irrigation showed a significant difference (p < 0.05), whereas all other treatments and plant species exhibited non-significant differences (p > 0.05), as shown in **Figure 1**.

The weight per plant of both plant species showed significant effects (p < 0.01) in all single-factor analyses, including water treatment, planting method, and species. Among the interaction effects, only the interaction between water treatment and species was significant (p < 0.01), whereas all other interactions were non-significant (p > 0.05). However, the interaction analysis revealed several significant differences among treatment combinations (p < 0.05), as shown in **Figure 1**. Under inter-cropping conditions, Sesbania maintained a higher plant weight than HS under both water treatments.

The yield of both plant species showed significant effects (p < 0.01) in all single-factor analyses, including water treatment, planting method, and species. Interaction effects between two factors were also significant (p < 0.05), whereas the three-factor interaction was non-significant (p > 0.05). As illustrated in **Figure 1**, Sesbania maintained a higher yield than HS under inter-cropping conditions.

### Leaf chlorophyll content and chlorophyll fluorescence

**Figure 2:**
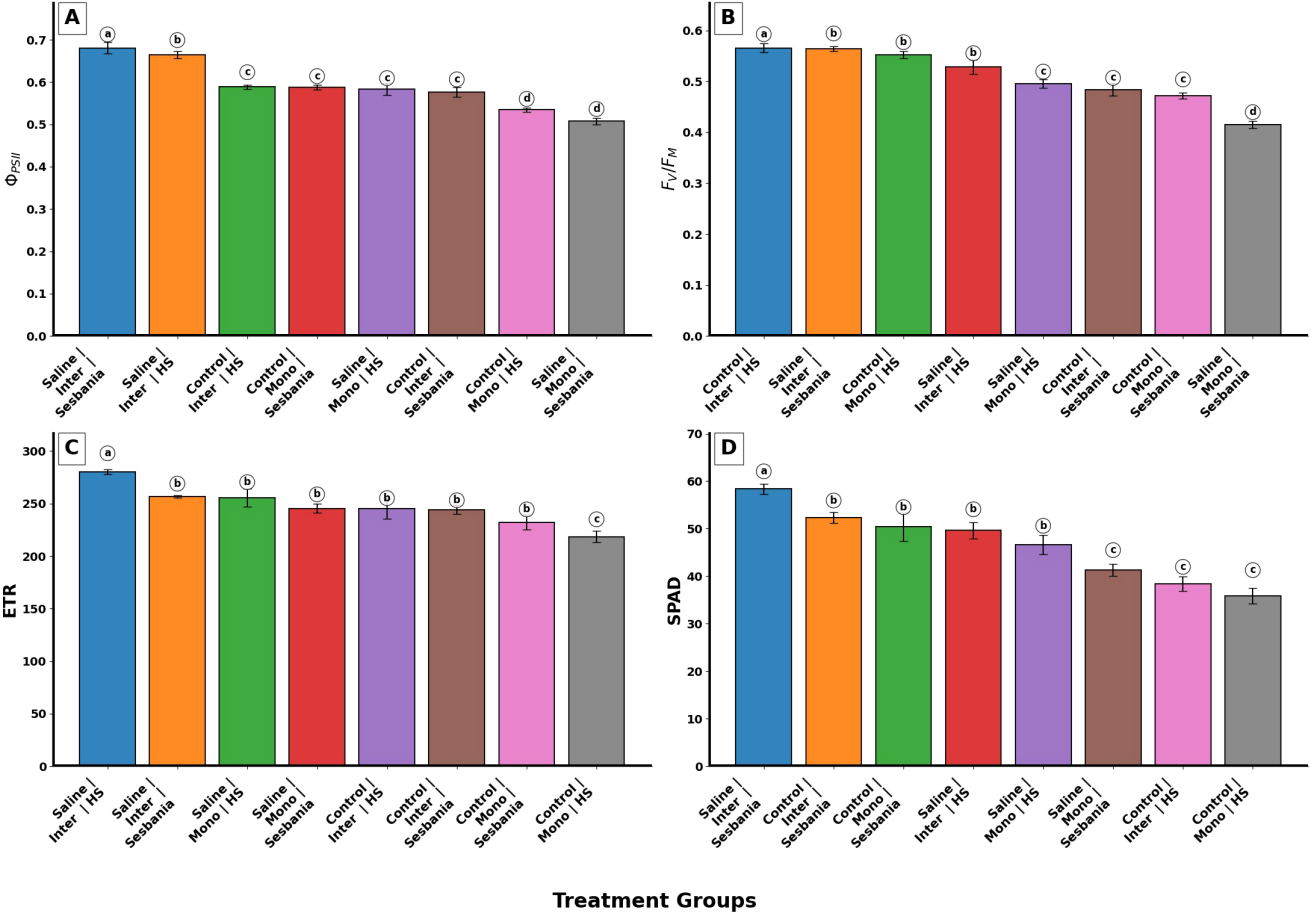
Effects of planting methods and irrigation on leaf chlorophyll content and chlorophyll fluorescence parameters of both plant species: **(A)** effective quantum yield of photosystem II (Φ_PSII_), **(B)** maximal photochemical efficiency of photosystem II (F_V_/F_M_), **(C)** electron transport rate (ETR), and **(D)** leaf chlorophyll content measured as SPAD (soil plant analysis development) values. Different letters indicate the significant differences among treatment interactions (p < 0.05). Inter, inter-cropping; Mono, mono-cropping; HS, *Hedysarum scoparium* Fisch. et Mey.; SPAD, leaf chlorophyll content.

A three-way ANOVA was performed to evaluate the effects of planting method, species, and water treatment, as well as their interactions, on the effective quantum yield of photosystem II (Φ_PSII_). The ANOVA results showed that Φ_PSII_ was significantly affected by interaction effects (p < 0.01), whereas the main effect of water treatment was non-significant (p > 0.05). As shown in **Figure 2**, Sesbania exhibited significantly higher Φ_PSII_ values than HS under inter-cropping conditions under both control and saline irrigation (p < 0.05).

The maximal photochemical efficiency of photosystem II (F_V_/F_M_) showed significant main effects (p < 0.01) and significant interaction effects (p < 0.01), with the exception of water treatment, which had a non-significant effect (p > 0.05). The interaction analysis revealed that Sesbania exhibited higher F_V_/F_M_ values as compared to HS under inter-cropping. In contrast, under mono-cropping with saline irrigation, HS exhibited higher F_V_/F_M_ values than Sesbania, as shown in **Figure 2**.

ETR showed significant interaction effects for planting method × species (p < 0.01) and water treatment × species (p < 0.01). Significant main effects were also observed for water treatment (p < 0.01) and planting method (p < 0.01). Under saline irrigation and inter-cropping conditions, HS exhibited significantly higher ETR values than Sesbania; however, this difference was not statistically significant (p > 0.05). In contrast, under control irrigation, Sesbania showed slightly higher ETR values than HS, but this difference was also non-significant (p > 0.05), as shown in **Figure 2**.

SPAD showed significant main effects and interaction effects (p < 0.01) in the ANOVA, except for planting method, which had a non-significant effect (p > 0.05). Under saline irrigation and inter-cropping conditions, SPAD values were significantly higher (p < 0.05) in Sesbania as compared to HS, as shown in **Figure 2**.

### Photosynthesis pigment

**Figure 3:**
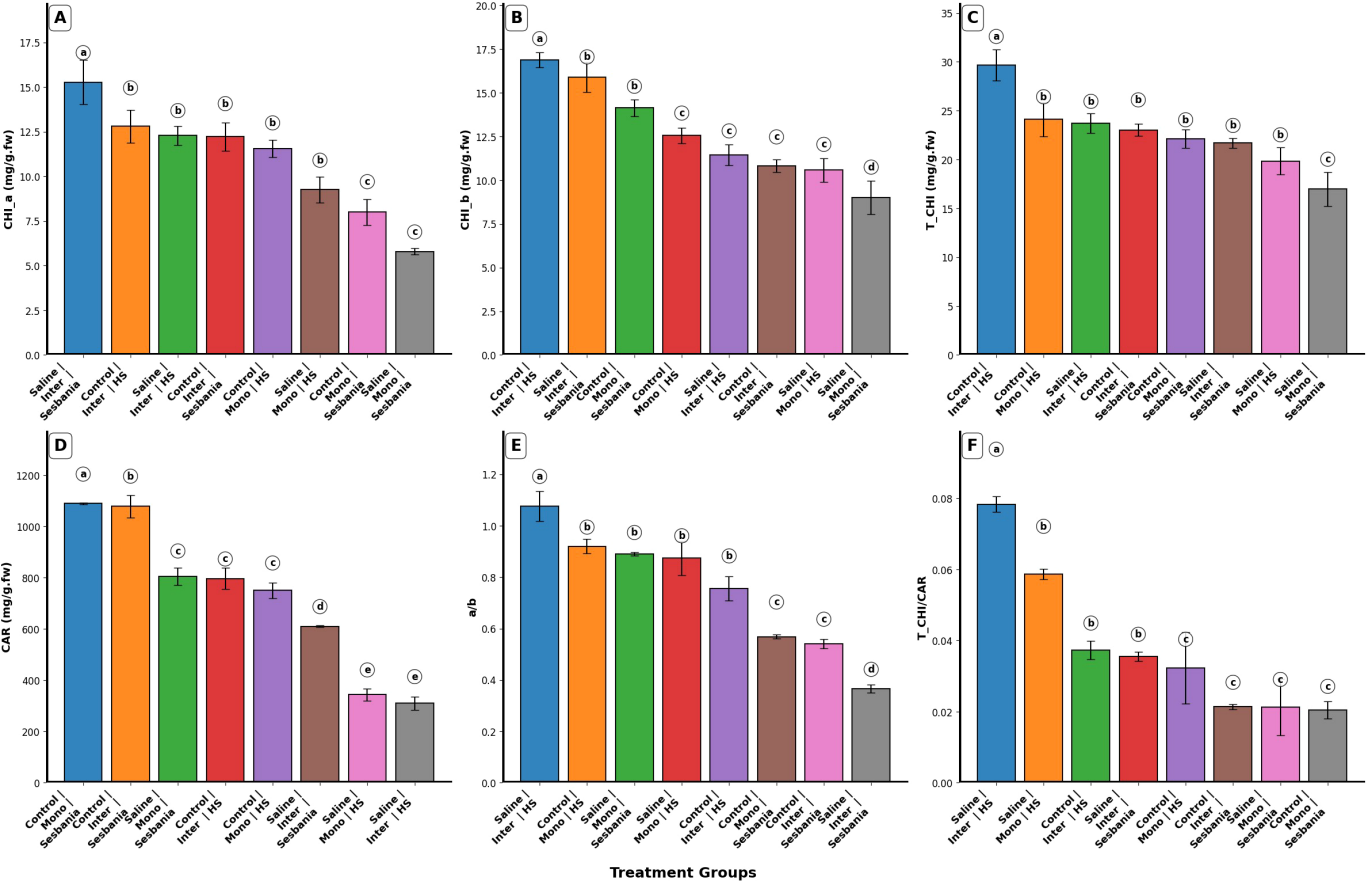
Effects of planting methods and irrigation on photosynthetic pigments of both plant species: **(A)** chlorophyll *a* (CHI_a), **(B)** chlorophyll *b* (CHI_b), **(C)** total chlorophyll (T_CHI), **(D)** carotenoids (CAR), **(E)** *a*/*b* ratio (chlorophyll *a*/chlorophyll *b*), and **(F)** T_CHI/CAR (total chlorophyll/carotenoids). Different letters indicate the significant differences among treatment interactions (p < 0.05). Inter, inter-cropping; Mono, mono-cropping; HS, *Hedysarum scoparium* Fisch. et Mey.

Variations in CHI_a, CHI_b, total chlorophyll (T_CHI), *a*/*b*, CAR, and T_CHI/CAR were observed under different planting methods and different water treatments, as shown in **Figure 3**. CHI_a, CHI_b, and T_CHI were significantly (p < 0.001) affected by water treatment, whereas planting method had no significant effect (p > 0.05). Significant interaction effects for CHI_a and T_CHI were observed for water treatment × species (p < 0.05) and planting method × species (p < 0.05). In contrast, CHI_b showed significant interaction effects for planting method × species (p < 0.01) and planting method × species × water treatment (p < 0.05). CAR, *a*/*b* ratio, and T_CHI/CAR were significantly affected by all single factors (water treatment, planting method, and species) (p < 0.01), except T_CHI/CAR, which showed a non-significant effect of planting method (p > 0.05). Furthermore *a*/*b* ratio showed significant effects across all interaction terms (p < 0.01), whereas CAR and T_CHI/CAR showed non-significant interaction effects for water treatment × species, plant method × species, and water treatment × plant method × species (p > 0.05). Compared with control conditions, both species exhibited marked variations in CHI_a, CHI_b, and T_CHI under saline irrigation across planting methods. Sesbania showed significantly higher values of CHI_a and CHI_b (p < 0.05) as compared with HS under inter-cropping and saline irrigation, as shown in **Figure 3A**. Both plant species showed variations in CAR content under both planting methods; however, under inter-cropping and saline irrigation, Sesbania exhibited higher CAR values as compared with HS, as shown in **Figure 3**. Under inter-cropping and saline irrigation, the *a*/*b* ratio was significantly (p < 0.05) higher in HS than in Sesbania, as shown in **Figure 3**. The T_CHI/CAR also showed significant interaction trends (p < 0.05), with HS exhibiting significantly higher values than Sesbania (p < 0.05), as shown in **Figure 3**.

### Oxidative stress markers and antioxidant

**Figure 4:**
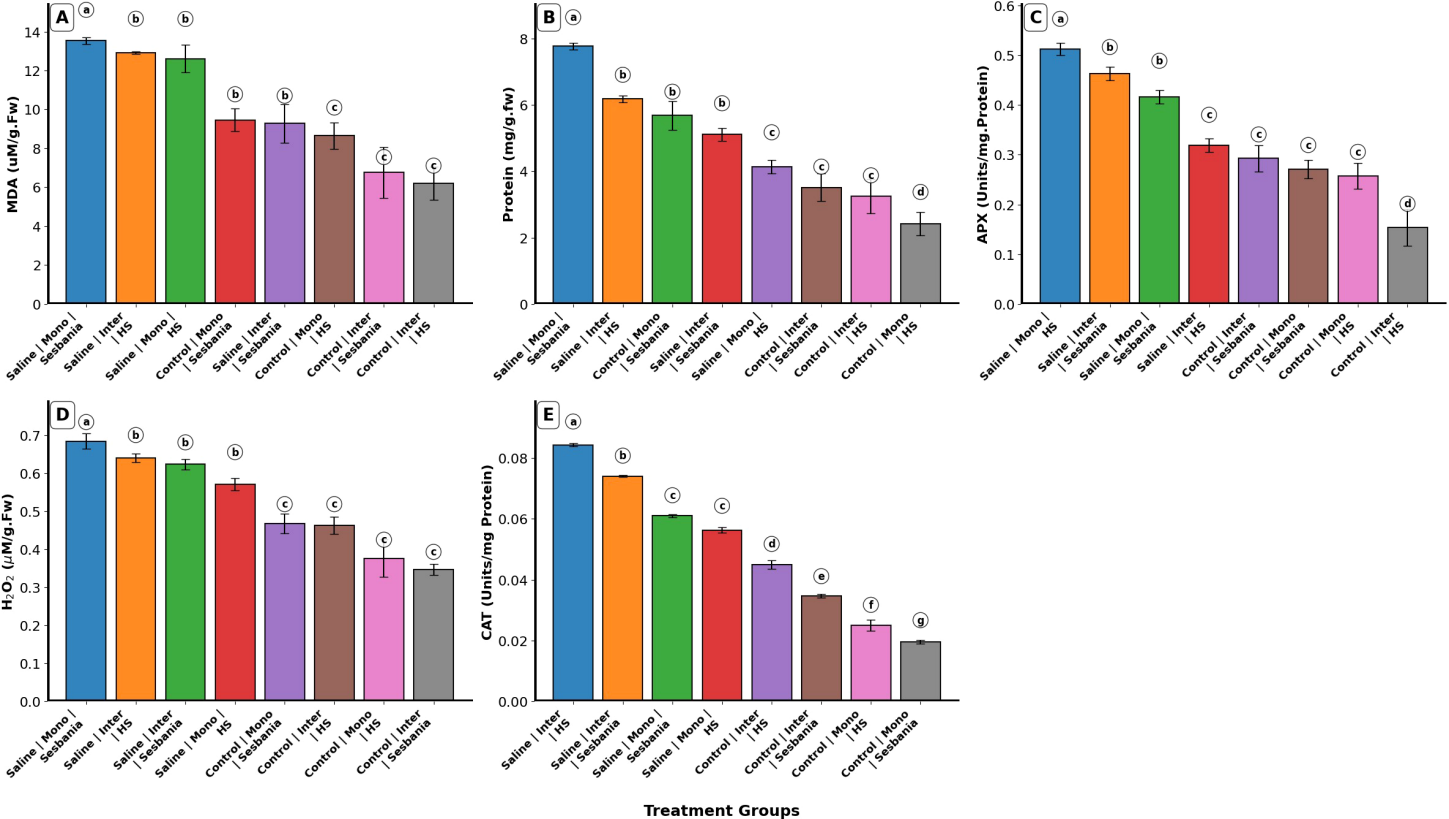
Effects of planting methods and irrigation on oxidative stress markers and antioxidants of both plant species: **(A)** malondialdehyde (MDA) content, **(B)** soluble protein content (Protein), **(C)** ascorbate peroxidase activity (APX), **(D)** hydrogen peroxide (H_2_O_2_), and **(E)** catalase activity (CAT). Different letters indicate the significant differences among treatment interactions (p < 0.05). Inter, inter-cropping; Mono, mono-cropping; HS, *Hedysarum scoparium* Fisch. et Mey.

Protein content and APX activity showed significant main effects in all single-factor analyses, including water treatment, planting method, and species (p < 0.05). MDA showed significant interaction effects for planting method × species (p < 0.05) and planting method × species × water treatment (p < 0.05). Under inter-cropping and saline irrigation, HS exhibited significantly higher MDA values than Sesbania (p < 0.05), whereas under control irrigation and inter-cropping, Sesbania showed higher but non-significant (p > 0.05) MDA values compared to HS, as shown in **Figure 4**. HS maintained similar MDA levels under both water treatment and inter-cropping. Protein content showed a significant interaction effect only for planting method × species (p < 0.001), while all other interactions were non-significant (p > 0.05). Protein content increased under saline irrigation of both plant species. Under saline irrigation and inter-cropping, HS exhibited higher but non-significant protein content than Sesbania (p > 0.05). In contrast, under all other treatments in both mono-cropping and inter-cropping, Sesbania showed significantly higher protein content than HS (p < 0.05), as shown in **Figure 4**.

For APX activity, a significant interaction effect was observed only for planting method × species (p < 0.01). APX activity increased significantly (p < 0.05) under saline irrigation in both plant species, as shown in **Figure 4**. Under saline irrigation and inter-cropping conditions, Sesbania exhibited significantly higher APX activity than HS (p < 0.05). H_2_O_2_ showed a significant main effect of water treatment (p < 0.001) and a significant interaction effect of planting method × species (p < 0.01). H_2_O_2_ levels increased significantly (p < 0.05) under both planting methods and saline irrigation in both plant species. Under mono-cropping, Sesbania exhibited significantly (p < 0.05) higher values as compared to HS, whereas under inter-cropping and saline irrigation, HS showed higher but non-significant H_2_O_2_ levels compared with Sesbania (p > 0.05), as shown in **Figure 4**.

CAT activity showed significant main effects in all single-factor analyses (p < 0.001), as well as significant interaction effects (p < 0.05). CAT activity increased significantly (p < 0.05) under saline irrigation in both plant species. Under saline irrigation, CAT activity in HS was significantly higher (p < 0.05) than in Sesbania under inter-cropping conditions; however, under mono-cropping, Sesbania showed significantly higher CAT activity than HS (p < 0.05), as shown in **Figure 4**.

### Ion concentration

**Figure 5:**
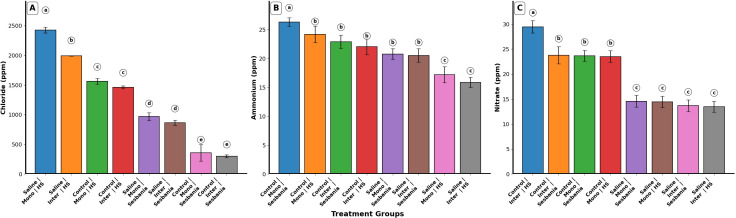
Effects of planting methods and irrigation on ion concentrations: **(A)** chloride, **(B)** ammonium, and **(C)** nitrate. Different letters indicate the significant differences among treatment interactions (p < 0.05). Inter, inter-cropping; Mono, mono-cropping; HS, *Hedysarum scoparium* Fisch. et Mey.

Water treatment and planting method had significant effects on ion concentrations in both plant species (p < 0.05). Chloride (Cl) and ammonium (NH_4_) showed significant main effects in all single-factor analyses (p < 0.05). However, NH_4_ exhibited no significant interaction effects (p > 0.05), whereas Cl showed significant interaction effects for water treatment × planting method (p < 0.05) and planting method × species (p < 0.01). In contrast, nitrate (NO_3_) showed a significant main effect only for water treatment (p < 0.001) and a significant interaction effect for water treatment × planting method (p < 0.05). Cl concentration increased significantly (p < 0.05) in both plant species under both planting methods and saline irrigation, as shown in **Figure 5**. Under saline irrigation and inter-cropping conditions, HS exhibited significantly higher Cl concentrations than Sesbania (p < 0.05).

NH_4_ and NO_3_ concentrations in both plant species decreased significantly under saline irrigation compared with control conditions across both planting methods (p < 0.05). Under both mono-cropping and inter-cropping, Sesbania exhibited significantly higher NH_4_ and NO_3_ concentrations than HS (p < 0.05), as shown in **Figure 5B**.

### Heat map and PCA

The heat map illustrates the response of the treatment group against different parameters. The vertical axis represents the treatment group, whereas the horizontal axis represents growth and stress parameters. Stress markers such as APX, MDA, and H_2_O_2_ showed strong stress responses under the combination of mono × saline irrigation × Sesbania. However, stress in Sesbania was reduced under inter-cropping × saline irrigation × Sesbania, indicating that HS alleviated the stress effects and allowed Sesbania to better sustain its growth. Photosynthesis pigment (CHI_a, CHI_b, and T_CHI) in Sesbania were reduced under saline irrigation in both planting methods, whereas HS maintained relatively stable photosynthetic pigment levels under these conditions. Growth traits, including PH, RL, weight per plant (WPP), and yield, were reduced in both plant species under saline irrigation across both planting methods, as shown in **Supplementary Figure S2**.

**Figure 6:**
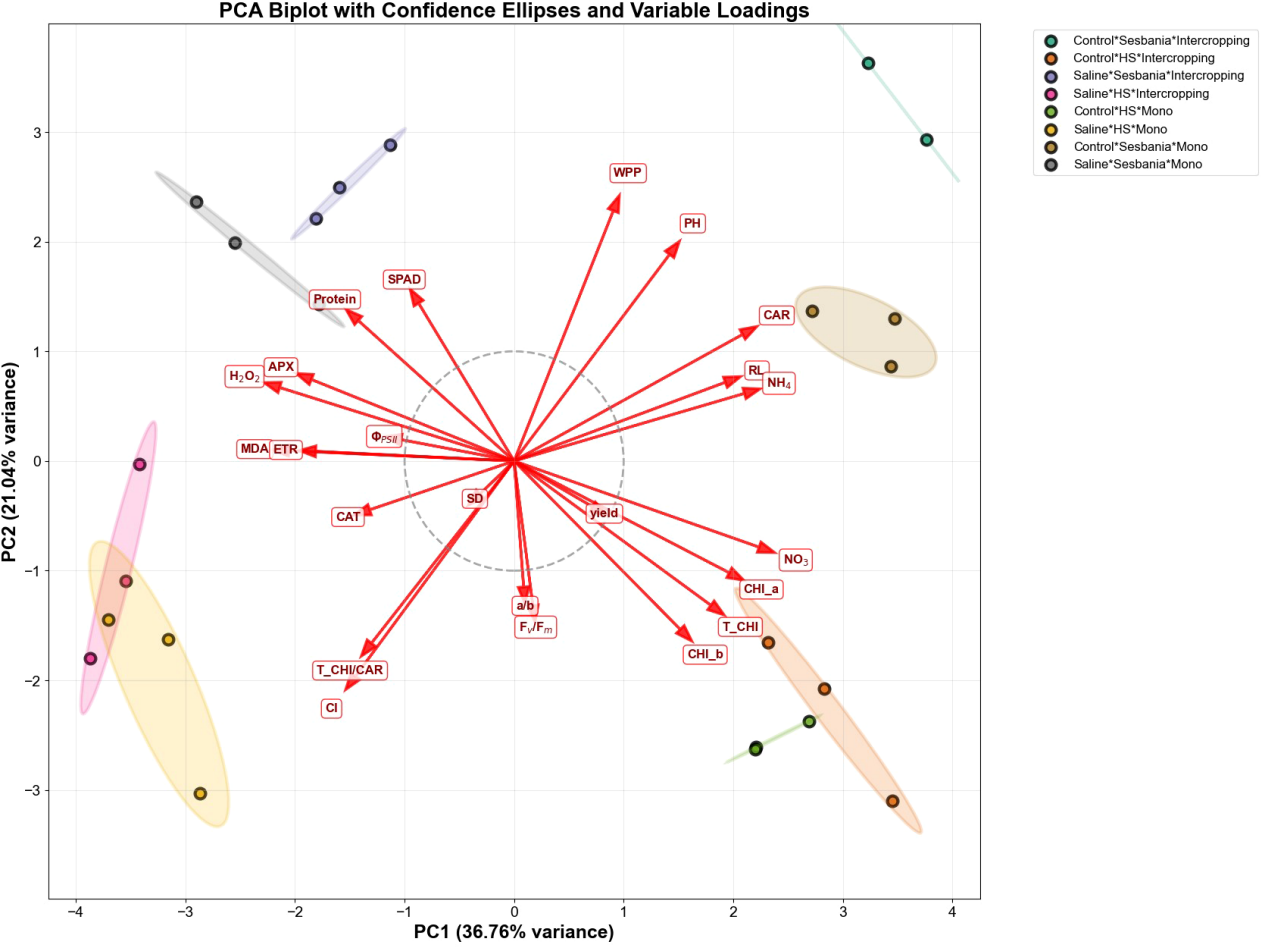
Principal component analysis (PCA) of both plant species under different planting methods.NO_3_, nitrate; CHI_b, chlorophyll *b*; Protein, soluble protein content; CHI_a, chlorophyll *a*; T_CHI, total chlorophyll; CAR, carotenoids; NH_4_, ammonium; RL, root length; PH, plant height; WPP, weight per plant; SD, stem diameter; F_V_/F_M_, maximal photochemical efficiency of photosystem II; CAT, catalase activity; T_CHI/CAR, total chlorophyll/carotenoids; Cl, chloride; SPAD, leaf chlorophyll content; Φ_PSII_, effective quantum yield of photosystem II; ETR, electron transport rate; APX, ascorbate peroxidase activity; MDA, malondialdehyde; H_2_O_2_, hydrogen peroxide.

PCA was performed on growth, physiological, and biochemical traits to assess the effects of different treatment combinations. The first two principal components (PC1 and PC2) collectively explained a substantial proportion of the total variance in the dataset (PC1 = 36.76%, PC2 = 21.04%), indicating that these components captured most of the variations among treatments, as shown in **Figure 6**. Both plant species under saline conditions formed distinct clusters compared with control treatments, indicating pronounced physiological shifts under stress conditions. The spatial separation of saline and control treatments along PC1 suggests that water treatment was the primary factor driving phenotypic variation. In contrast, the separation of HS and Sesbania along PC2 indicates species-specific adaptation mechanisms under stress conditions. Inter-cropping treatments showed lower stress effects compared with mono-cropping, suggesting that inter-cropping may provide partial protection against salt stress by improving the microclimate and resource availability.

## Discussion

### Role of halophyte crop inter-cropping in salinity amelioration and phytoremediation

Phytoremediation has emerged as a major research focus for managing salt-affected dry-land ecosystems in recent years (Sharma et al., 2023). Numerous studies have demonstrated that the use of halophytic plants in saline dry-land regions can significantly improve soil conditions and plant performance. However, information regarding the combined use of halophytes with salt-tolerant crops under inter-cropping systems in dry-land regions remains limited (Sofy et al., 2022, Azeem, Mai et al., 2025a). Such inter-cropping strategies hold substantial potential to mitigate salinity-induced stress in regions where excessive salt accumulation restricts crop growth and productivity (Azeem, Mai et al., 2023b, Azeem, Mai et al., 2025b). Halophytes possess a strong capacity to absorb and sequester salts from saline soils, thereby reducing salt toxicity, improving soil quality for associated cash crops, and contributing to the restoration of biodiversity in salt-affected ecosystems (Azeem et al., 2021b; Priya et al., 2023).

### Growth and yield response of Sesbania under inter-cropping in saline dry-land conditions

The experimental results demonstrated that the growth of the fodder crop Sesbania was significantly increased under saline irrigation when cultivated in an inter-cropping system. The PH of Sesbania was consistently higher under inter-cropping, whereas the RL of HS was markedly increased, indicating the development of an extensive root system under saline conditions. Such root proliferation likely enabled the halophyte to capture excess salt ions and access fresher water from deeper soil layers (Liu et al., 2025). The upward movement of this fresher water by HS may have contributed to the dilution of surface soil salinity, thereby creating a more favorable rhizosphere environment for Sesbania (Liu et al., 2025).

**Figure 7:**
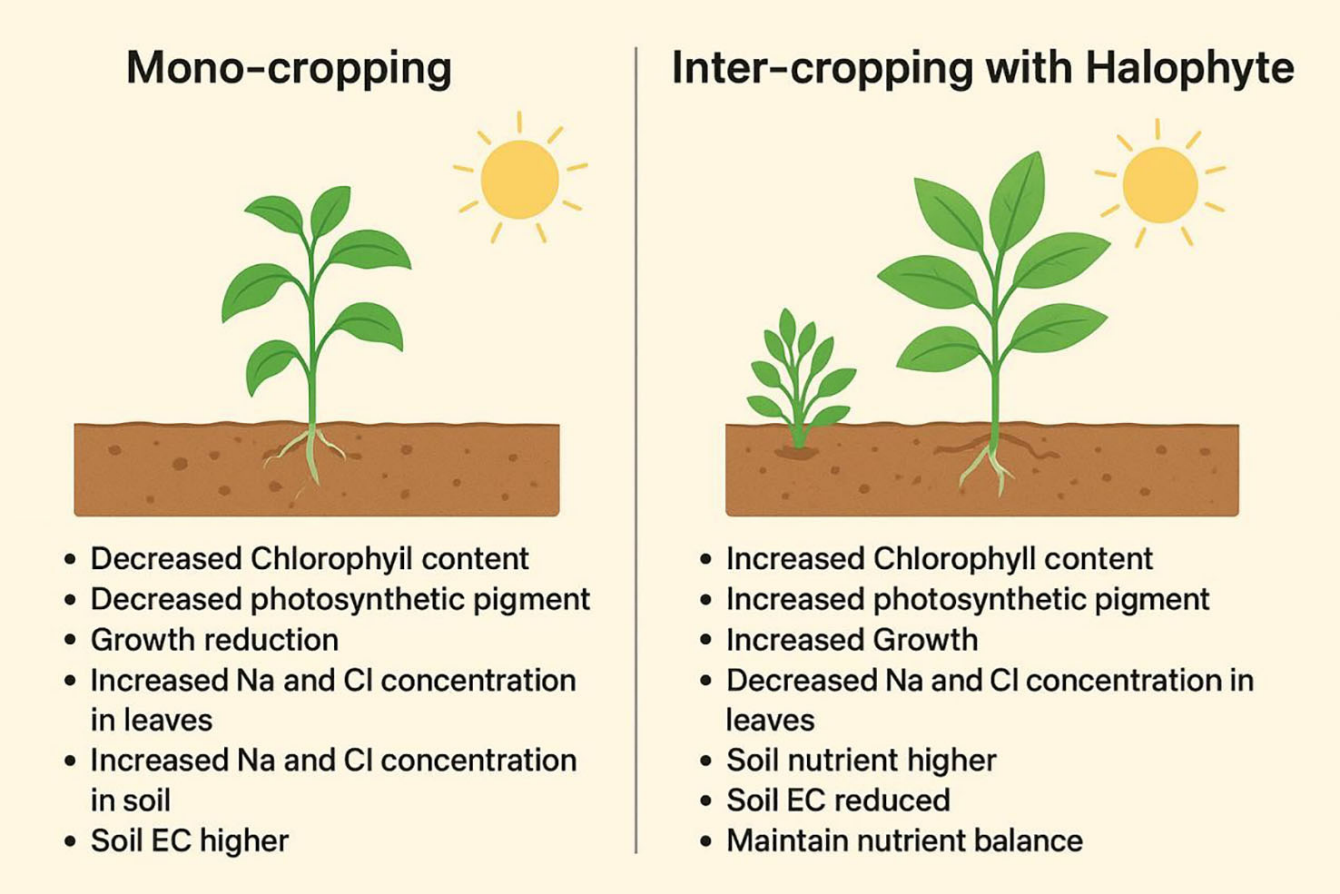
Main changes in Sesbania under mono-cropping and inter-cropping in dry-land region under saline irrigation. Na, sodium; Cl, chloride; EC, electrical conductivity.

Furthermore, inter-cropping significantly improved Sesbania productivity compared with mono-cropping, suggesting that HS effectively moderated salt accumulation at the soil surface and reduced the uptake of toxic ions such as Na and Cl by the companion crop (Bo et al., 2024). In addition to regulating soil salinity, HS under inter-cropping conditions likely enhanced nutrient absorption and utilization efficiency, which contributed to the improved crop performance across irrigation salinity levels in dry-land environments (Ren et al., 2023). These findings are consistent with previous reports demonstrating that halophyte-based inter-cropping systems can enhance nutrient availability and rhizosphere functioning in associated crops (Wang et al., 2022; Dai et al., 2025). Improved nutrient availability under saline irrigation has been shown to alleviate salinity-induced growth constraints in dry-land ecosystems (Hamed et al., 2021). In the present study, yield was positively associated with PH, and yield estimates based on weight per plant further supported the beneficial role of inter-cropping in enhancing crop performance under saline conditions. The comparative growth and yield responses of Sesbania under mono-cropping and inter-cropping systems across saline irrigation treatments are shown in **Figure 7**.

Both Sesbania and HS exhibited contrasting yet complementary adaptive responses across salinity levels. Sesbania, a moderately salt-tolerant crop, showed reductions in growth and yield under mono-cropping at higher salinity, primarily due to excessive Na, Cl accumulation, and increased osmotic stress (Sharavdorj et al., 2024). In contrast, HS maintained relatively stable growth across the salinity gradients by enhancing root elongation and salt sequestration capacity. Under inter-cropping conditions, HS mitigated salinity stress by lowering salt concentrations in the shared rhizosphere, thereby creating a more favorable soil environment for Sesbania. As a result, Sesbania sustained improved growth and yield even under high salinity levels (Guerchi et al., 2024). This synergistic response demonstrates the functional complementarity of halophytes and legumes in stabilizing crop performance across variable saline regimes in dry lands (Hao et al., 2025; Wang et al., 2025).

### Nutrient uptake, ionic balance, and nitrogen cycling under inter-cropping

Salt stress restricts plant growth through multiple mechanisms, with damage to leaf cells being one of the primary effects (Azeem et al., 2020a, 2020). Excessive accumulation of Na and Cl disrupts cellular homeostasis, initially inducing osmotic stress, subsequently impairing nutrient balance and redox stability. Similar growth reductions in Sesbania under saline conditions have been widely reported in dry-land systems, particularly under mono-cropping where salt accumulation remains unchecked (Genzel et al., 2021, Azeem, Mai et al., 2024b). Although Sesbania varieties possess regulatory mechanisms such as controlled Na uptake, improved K partitioning, and enhanced antioxidant capacity, prolonged exposure to high salinity can still induce physiological drought. This condition restricts water uptake and reduces biomass production (Khan et al., 2024). Rather than reiterating stress indicators, the present findings suggest that inter-cropping altered the physiological context in which stress responses occurred. Improved photosynthetic functionality under inter-cropping reflected a more favorable internal environment rather than simple stress avoidance (Khan et al., 2022). This response is closely associated with enhanced nitrogen availability, as legumes such as Sesbania contribute to soil N enrichment, which supports chlorophyll biosynthesis and maintains photosystem integrity (Nikam et al., 2024; Yu et al., 2025). Concurrently, HS modified the rhizosphere through salt sequestration, thereby limiting ionic interference with nutrient assimilation. Similar halophyte-mediated improvements in photosynthetic stability have been reported in inter-cropping systems under saline dry-land conditions (Simon and Yusuf, 2020; Sun et al., 2025). This functional complementarity enabled both species to maintain stable electron transport processes and photosynthetic pigment composition, supporting sustained biomass production under salinity stress.

In dry-land ecosystems, the combined effects of salinity and sandy soils accelerate oxidative stress through excessive reactive oxygen species (ROS) formation, leading to membrane lipid peroxidation and protein degradation (Aktaş et al., 2005; Hlas et al., 2024). While elevated antioxidant enzyme activity under saline irrigation represents a common stress response, inter-cropping further strengthened these protective mechanisms (Azeem et al., 2017, 2025a, 2025b). Enhanced activities of CAT and APX, together with increased total protein content, reduced H_2_O_2_ and MDA accumulation, thereby limiting cellular damage (Azeem et al., 2021a, Azeem, Mai et al., 2025a). Similar antioxidant reinforcement has been observed in halophyte-assisted inter-cropping systems, highlighting their role in buffering oxidative stress rather than merely inducing stress-related responses (Boorboori and Zhang, 2025). This suggests that inter-cropping activates a coordinated defense strategy that integrates osmotic adjustment with improved ROS detoxification (Nasreldein et al., 2021).

Nutrient balance and uptake are among the most limiting factors for crop development under salt stress (Pitaloka and Usmadi, 2023). In the present study, mono-cropping under saline irrigation intensified Cl accumulation while reducing NH_4_ and NO_3_ availability in Sesbania, reflecting impaired nutrient acquisition under ionic stress. In contrast, inter-cropping substantially improved nutrient uptake by modifying the rhizosphere environment (Mathur and Mathur, 2024). HS effectively removed excess salts from the soil solution, thereby reducing ion toxicity and enhancing nutrient availability for Sesbania. Comparable improvements in nutrient balance have been reported in halophyte-assisted inter-cropping systems involving legumes [*Sesbania cannabina* (Retz.) Poir and *Medicago sativa* L.] (Guerchi et al., 2024; Huang et al., 2024) and cereal crops (*Zea mays* L. and *Triticum aestivum* L.) under saline dry-land conditions (Wang et al., 2022). Additionally, inter-cropping influenced nitrogen dynamics by stabilizing NH_4_ and NO_3_ pools, reducing losses through volatilization and denitrification commonly accelerated under high salinity (Li et al., 2020, KhavaninZade et al., 2022). Overall, halophyte-based inter-cropping enhanced Na sequestration by HS. This process simultaneously improved nutrient availability and utilization efficiency in Sesbania, providing a mechanistic basis for improved performance under saline dry-land conditions.

## Conclusion

In conclusion, this study demonstrates that inter-cropping Sesbania with HS enhances plant growth and fodder yield under saline irrigation in dry-land conditions. These benefits are associated with reduced salt accumulation in leaf tissues and improved physiological and biochemical performance. Overall, halophyte-based inter-cropping represents an eco-friendly and cost-effective approach to improve productivity on salt-affected soils and promote the efficient use of saline water resources. The practical adoption of this strategy relies on the selection of locally adapted halophytic species, appropriate planting configurations, and effective soil and water management practices. However, its performance may vary with salinity intensity, soil texture, water availability, and land management conditions, particularly in severely degraded soils. Therefore, further research is required to support long-term field validation and optimize inter-cropping designs. Additional studies should evaluate the applicability of this approach across different crops and salinity gradients to facilitate wider implementation.

## Data Availability

The raw data supporting the conclusions of this article will be made available by the authors, without undue reservation.
