# Supplementary material for: Mitigation of salinity stress in Sesbania through inter-cropping with halophyte Hedysarum scoparium in dry-land conditions
Source: Front Plant Sci. 2026 Mar 11;17:1756353. doi: 10.3389/fpls.2026.1756353 (PMC13014542; doi:10.3389/fpls.2026.1756353)
Supplement: Supplementary file 1 [file Supplementaryfile1.docx]

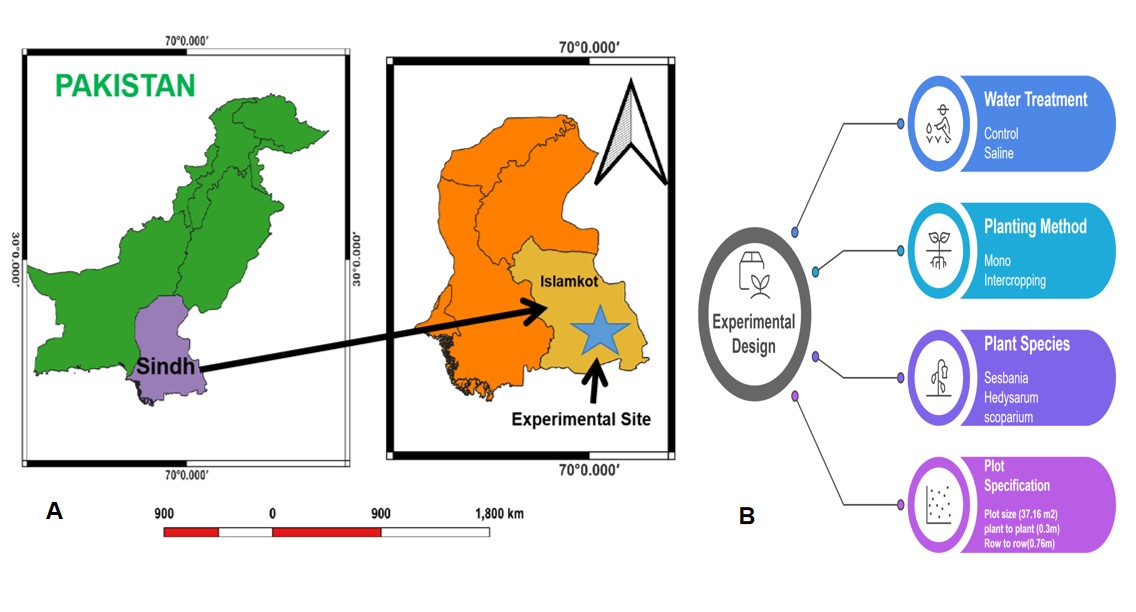
**Figure S1** (A) Study site location and (B) Experimental design.


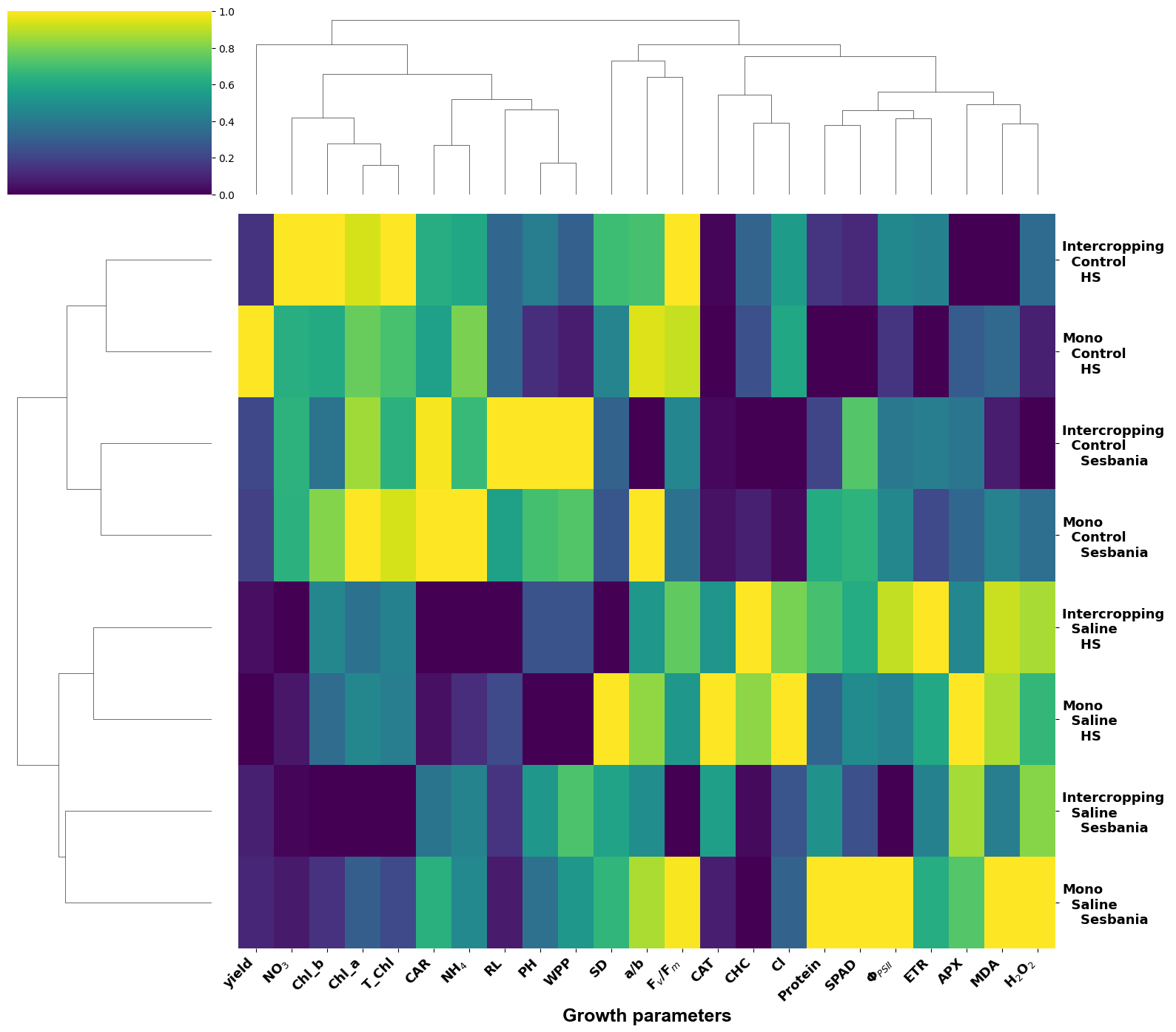


**Figure S2** Heat map with dendrogram between morpho-physiological, biochemical attributes and ionic contents of both plant species under different planting methods. Note: Nitrate (NO_3_), Chlorophyll b (Chl_b), Chlorophyll a (ChI_a), Total chlorophyll (T_ChI), Carotenoids (CAR), Ammonium (NH_4_), Root length (RL), Plant height (PH), Weight per plant (WPP), Stem diameter (SD), Maximal Photochemical Efficiency of Photosystem II (F_V_/F_M_), Catalase (CAT), Total chlorophyll/Carotenoids (CHC). Chloride (CI), Leaf chlorophyll content (SPAD), Effective quantum yield of photosystem II (Φ_PSII_), Electron transport rate (ETR), Ascorbate peroxidase (APX), Malondialdehyde Content (MDA), Hydrogen per oxide (H_2_O_2_), inter = inter-cropping, Mono= mono-cropping, HS = *Hedysarum scoparium Fisch. et Mey.*
